# CT2A Cell Viability Modulated by Electromagnetic Fields at Extremely Low Frequency under No Thermal Effects

**DOI:** 10.3390/ijms21010152

**Published:** 2019-12-24

**Authors:** Olga García-Minguillán, Raquel Prous, Maria del Carmen Ramirez-Castillejo, Ceferino Maestú

**Affiliations:** 1 Escuela Técnica Superior de Ingenieros de Telecomunicación, Universidad Politécnica de Madrid, 28040 Madrid, Spain; olga.garcia-minguillan@ctb.upm.es (O.G.-M.); r.prous@alumnos.upm.es (R.P.); 2 CTB (CTB-UPM) Centro de Tecnología Biomédica, Universidad Politécnica de Madrid, 28223 Pozuelo de Alarcón, Spain; 3 ETSIAAB. Dpto Biotecnologia-BV. HST group, Universidad Politécnica de Madrid, 28040 Madrid, Spain; mariadelcarmen.ramirez@upm.es; 4 CIBER-BBN Centro de Investigación Biomédica en Red, 28029 Madrid, Spain

**Keywords:** ELF-EMF, CT2A, cell viability, HSP90

## Abstract

The effects produced by electromagnetic fields (EMFs) on human beings at extremely low frequencies (ELFs) have being investigated in the past years, across in vitro studies, using different cell lines. Nevertheless, the effects produced on cells are not clarified, and the cellular mechanisms and cell-signaling processes involved are still unknown. This situation has resulted in a division among the scientific community about the adequacy of the recommended level of exposure. In this sense, we consider that it is necessary to develop long-term exposure studies and check if the recommended levels of EMFs are under thermal effects. Hence, we exposed CT2A cells to different EMFs at different ELFs at short and long times. Our results showed frequency dependence in CT2A exposed during 24 h to a small EMF of 30 μT equal to those originated by the Earth and frequency dependence after the exposure during seven days to an EMF of 100 µT at different ELFs. Particularly, our results showed a remarkable cell viability decrease of CT2A cells exposed to EMFs of 30 Hz. Nevertheless, after analyzing the thermal effects in terms of HSP90 expression, we did not find thermal damages related to the differences in cell viability, so other crucial cellular mechanism should be involved.

## 1. Introduction

Electromagnetic fields (EMFs) are the physical combination of electric fields and magnetic fields, which constitutes one of the four fundamental forces [1]. They are created both naturally by the earth and artificial by the human [2]. The principal parameter that defines them is their frequency, based on which they are classified as follows: static fields (0 KHz), extremely low frequency (ELF)-EMFs (<300 Hz), and radiofrequency (RF)-EMFs (>300 Hz). Nowadays, we are exposed continuously to EMFs which can produce biological effects both at ELFs and microwave frequencies. For that reason, to ensure the maximum security, the European Council stablished in 1999 an EMF of $\frac{5000}{frequency}$ µT as a recommended level for ELFs between 8 and 25 Hz and an EMF of $\frac{5}{frequency}$ µT for ELFs from 25 up to 800 Hz [3].

Notwithstanding, from 2000 to 2004, the adequacy of the recommended levels was studied by the joint research Reflex project, where, among other results, it was showed that effects of EMF exposure on cell viability depend on the cell line [4]. Moreover, in a recent in vitro study of the effects of ELF-EMF focused on glioblastomas we have also found cell viability dependence on frequency [5].

Nevertheless, the mechanisms involved were not clarified being suggesting by previous studies that EMF effects are related to voltage-gated calcium channels (VGCCs) [6] due to the increase of intracellular Ca^2+^ after the exposure [7,8,9,10]. Calcium ions play a crucial role in cell signaling since they act as principal second messengers in cell transduction [11,12]. Usually, intracellular Ca^2+^ is stored in organelles entering inside the cells by calcium channels and being removing from them by transport proteins. The increase of intracellular Ca^2+^ after the exposure to EMF also involves the increase of nitric oxide levels, which have been suggested in a previous study to produce both therapeutic and pathophysiological effects on cells [13].

The difference in the effects produced by an increase of Ca^2+^ after the exposure to an EMF in terms of cell viability is not properly understood, being necessary to complement the results available with the analysis of other fundamental parameters, like cell and heat damage, cell death, and cell proliferation, among others. Until now, most studies have analyzed those parameters in terms of expression of p53/p21 for the cellular damage [14,15,16], caspase 3 as an indicator of apoptosis [17,18], and heat-shock proteins (HSPs) as an indicator of heat damage [19]. However, the results obtained in most vitro research used EMF biggest that the stablished by the European Commission [3,20,21] and in any of them have been considered long term exposition nor the effects produced by EMF between 26–60 µT [22] equal to the geomatic fields originated by the earth at the different peaks of the Schuman Resonance [23].

Therefore, with our research, we aimed to amplify the studies done before by covering the following points: (1) analyze the effects produced by an EMF equal to the geomatic field; (2) analyze the effects produced by a long-term exposure to ELF-EMFs under the recommend level; (3) check if the exposure to ELF-EMF under the recommend level involves heat effects on the cells.

## 2. Results

### 2.1. Effect of Natural EMF on Cell Viability

**Figure 1:**
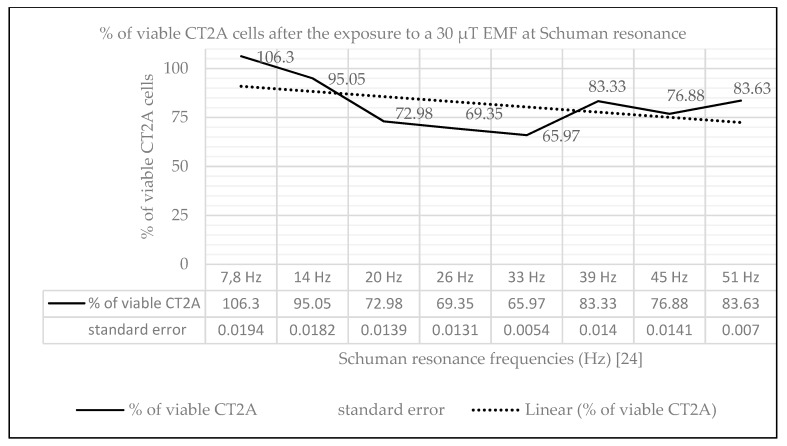
Graphical representation of % of viable CT2A cells after the exposure during 24 h to an EMF of 30 µT at the different frequencies of the Schuman resonance [23]: 7.8, 14, 20, 26, 33, 39, 45, and 51 Hz.

The effect produced on cell viability of CT2A cells by a 30 µT EMF equivalent to that produced naturally by the earth [23] at the Schuman resonances [24] was analyzed by XTT assay (Figure 1). Our results show at the main frequency of 7.8 Hz a small increase in cell viability (6.3%, *p* = 0.183). In the rest of the frequencies of the Schuman resonance analyzed it was archived the following decrease in cell viability with respect the control group: 14 Hz (4.95%, *p* = 0.231); 20 Hz (27.02%, *p* = 0.0006); 26 Hz (31.65%, *p* = 0.000014); 33 Hz (34.03%, *p* = 0.000012); 39 Hz (16.67%, *p* = 0.014); 45 Hz (23.12%, *p* = 0.004347); and 51 Hz (16.37%, *p* = 0.007).

### 2.2. Effects of Long-Term and Intermittent Exposure to a 100 µT EMF at Different ELF

**Figure 2:**
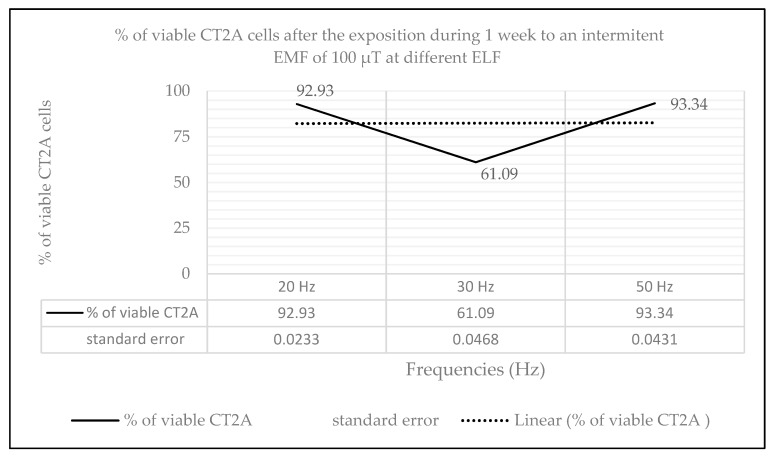
Graphical representation of % of viable CT2A cells after the exposure during seven days to an intermittent EMF of 100 µT at different ELFs: 20, 30, and 50 Hz.

The effect on CT2A cell viability after the exposure during 7 days to a 100 μT intermittent EMF (12 h coils on/12 h coils off per day) at different ELFs was measured by XTT assay (Figure 2). The results obtained show a slight decrease in cell viability at 20 Hz (6.66%, *p* = 0.124) and at 50 Hz (7.07%, *p* = 0.224). However, after the exposure to an intermittent EMF at 30 Hz it was obtained a bigger reduction in cell viability (38.91%, *p* = 0.0005) similar to our results obtained in previous studies at short term exposure [5].

### 2.3. Heat Effects Produced of CT2A Exposed to ELF-EMF of 100 µT

**Figure 3:**
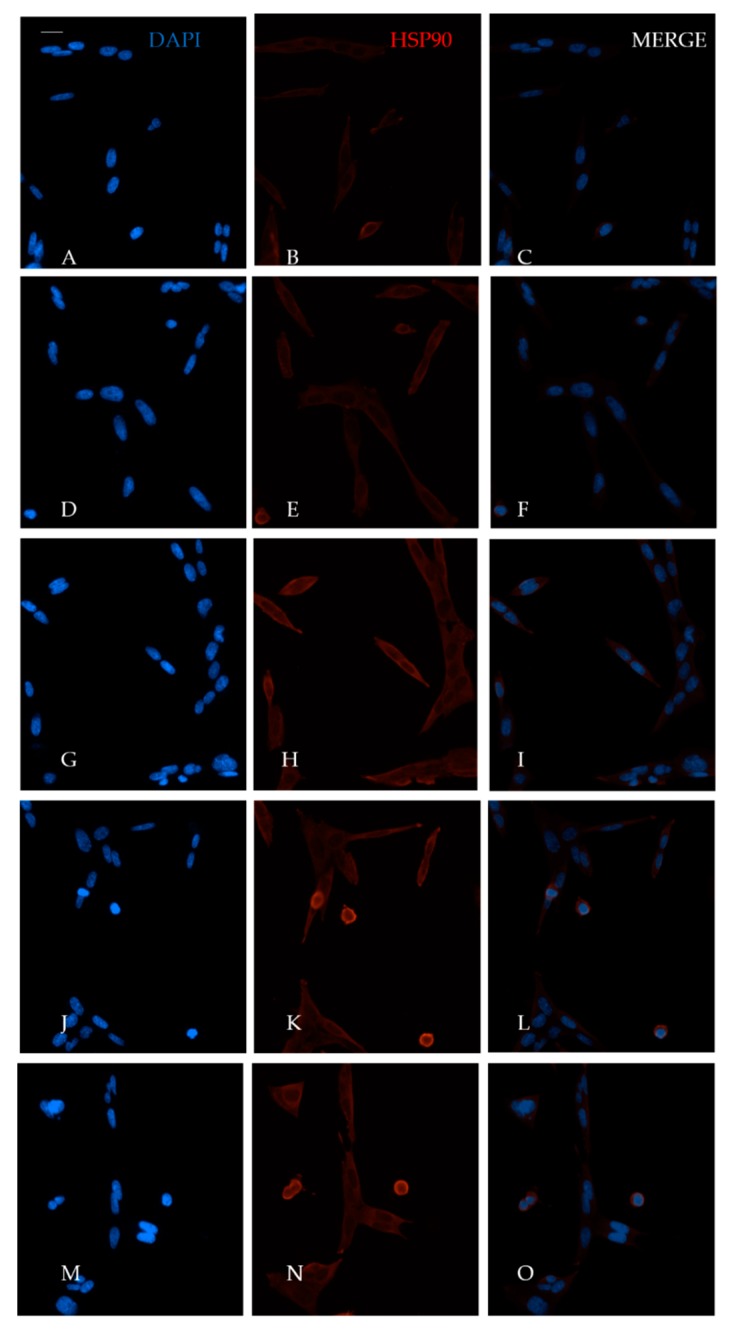
Immunofluorescence analysis of HSP90 expression in CT2A maintained during 1 h and 30 min at 5% CO_2_ and different temperatures (Microscope 20×): (**A**) 37 °C CT2A nuclei staining with DAPI; (**B**) 37 °C CT2A HSP90 expression; (**C**) merge of A and B; (**D**) 37.5 °C CT2A nuclei staining with DAPI; (**E**) 37.5 °C CT2A HSP90 expression; (**F**) merge of D and E. (**G**) 38 °C CT2A nuclei staining with DAPI; (**H**) 38 °C CT2A HSP90 expression; (**I**) merge of G and H. (**J**) 38.5 °C CT2A nuclei staining with DAPI; (**K**) 38.5 °C CT2A HSP90 expression; (**L**) merge of J and K. (**M**) 39 °C CT2A nuclei staining with DAPI; (**N**) 39 °C CT2A HSP90 expression; (**O**) merge of M and N.

**Figure 4:**
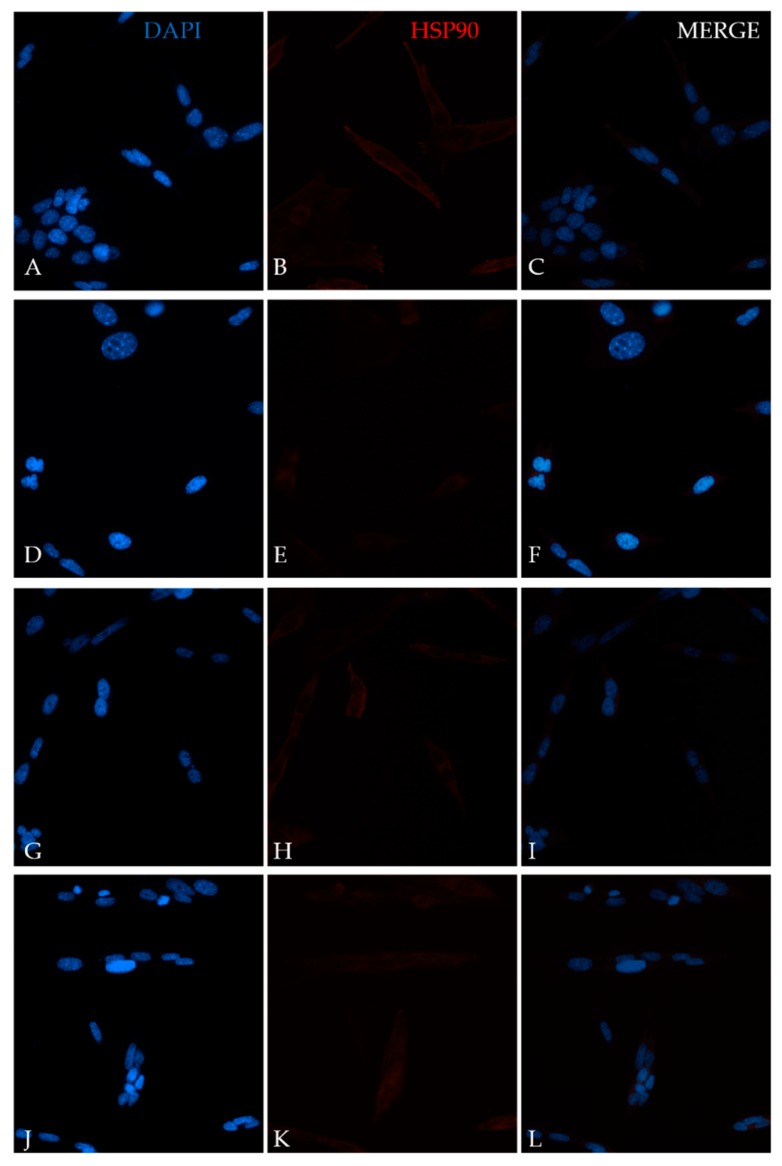
Immunofluorescence analysis of HSP90 expression in CT2A exposed to a 100 µT EMF (Microscope 20×): (**A**) CT2A control group (non-exposed), nuclei staining with DAPI; (**B**) CT2A control group (non-exposed) HSP90AB expression; (**C**) merge of A and B; (**D**) CT2A after the exposure to a 100 µT EMF at 20 Hz, nuclei staining with DAPI; (**E**) CT2A after the exposure to a 100 µT EMF at 20 Hz, HSP90AB expression; (**F**) merge of D and E; (**G**) CT2A after the exposure to a 100 µT EMF at 30 Hz, nuclei staining with DAPI; (**H**) CT2A after the exposure to a 100 µT EMF at 30 Hz, HSP90AB expression; (**I**) merge of G and H; (**J**) CT2A after the exposure to a 100 µT EMF at 50 Hz, nuclei staining with DAPI; **(K)** CT2A after the exposure to a 100 µT EMF at 50 Hz, HSP90AB expression; (**L**) merge of A and B.

**Figure 5:**
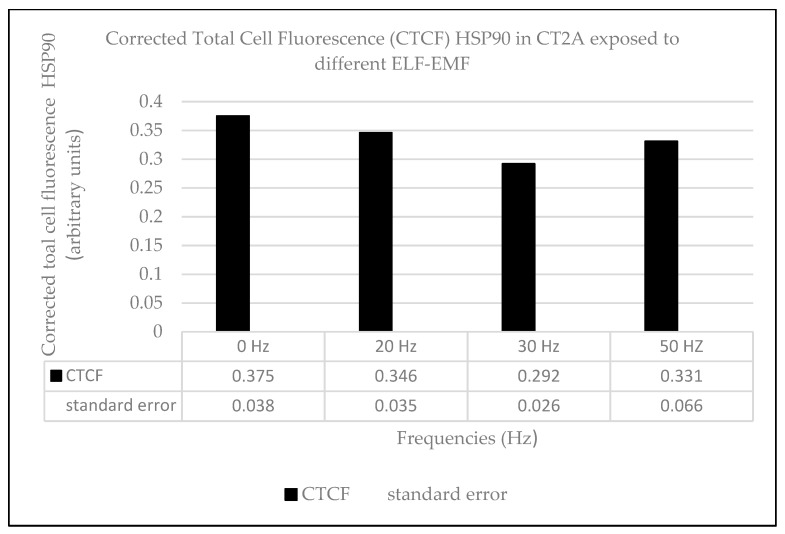
Corrected total cell fluorescence. Average corrected total cell fluorescence (CTCF) of HSP90 expression in CT2A control group (0 Hz, non-exposed) and CT2A cells after 24 h of exposure to a 100 µT EMF at different ELF: 20, 30, and 50 Hz.

The heat effects produced by ELF-EMFs on CT2A were studied by the immunocytochemical expression of the heat-shock protein HSP90. For that, CT2A cells were exposed during 24 h to a 100 µT EMF at 20, 30, and 50 Hz after which they were immunolabeled and analyzed by a fluorescent microscopy. The basal and positive expression of HSP90 in the CT2A was first identified in CT2A cells without exposure to different temperatures (37, 37.5, 38, 38.5, and 39 °C) obtaining an increase in the cytoplasmatic expression of HSP90 directly related to the increase in the temperature (Figure 3B,E,H,K,N). Thereby, according to our results in any of the frequencies analyzed were positive expression of HSP90 in the CT2A cells exposed to a 100 µT EMF (Figure 4E,H,K) with regards the control group (non-exposed) (Figure 4B). Moreover, there was no difference in the corrected total cell fluorescence (CTCF) of HSP90 in any of the frequencies analyzed (Figure 5, *p* with respect 37 °C: 0 Hz, *p* = 0.441; 20 Hz, *p* = 0.326; 30 Hz, *p* = 0.094; 50 Hz, *p* = 0.243). The slight expression showed in the CT2A cells exposed (Figure 4E,H,K) correspond to their natural basal expression at 37 °C (Figure 3B).

**Figure 6:**
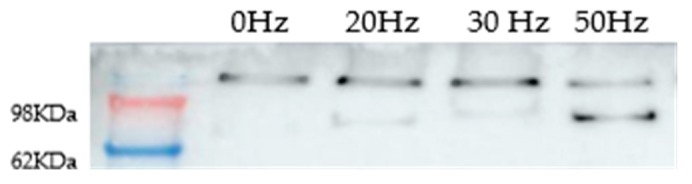
Western blot of HSP90 protein in CT2A non-exposed (0 Hz, Helmholtz coils off) and CT2A exposed during 24 h to an EMF of 100 µT at different ELF: 20, 30, and 50Hz.

On the other hand, the immunoblot showed a double band pattern (Figure 6) for all the frequencies analyzed (20, 30, and 50 Hz) and the control group of non-exposed CT2A cells (Helmholtz Coils off). The band between 98 and 68 KDa correspond to the native state of HSP90. This band, in the CT2A cells exposed to a 100 µT EMF at 50 Hz was bigger. Nevertheless, there was no difference in the total amount of HSP90 protein in the immunoblot between the control group and the CT2A exposed to the frequencies (*p* = 0.407, 20 Hz; *p* = 0.225, 30 Hz; *p* = 0.201, 50 Hz).

## 3. Discussion

The effects of ELF-EMF on cell viability were analyzed in the last years, using different cell lines and frequencies [3,21,24,25,26], revealing a dependence in the cell line, as well as we recently found dependence on frequency within the same line [5]. Particularly, in our previous study about the effect of ELF-EMF on glioblastomas, we found a reduction of cell viability from 13% up to almost 18% after a short time exposure to a 100 µT EMF at 30 Hz, and about an 8% increase in cell viability after the exposure to 50 Hz [5].

However, according to our present data, CT2A cells’ long-term exposure to a 100 µT intermittent EMF (12 h coils on/12 h coils off per day) at different ELF (Figure 2) showed relevant changes (*p* < 0.05) in cell viability with regard the control group at 30 Hz. In this sense, CT2A exposed to a 100 µT intermittent EMF at 30 Hz for 7 days showed a reduction of cell viability around 20% bigger than during the short time exposure [5]. On the other hand, the difference obtained in cell viability between the control group (coils off) and the CT2A exposed for seven days to a 100 µT intermittent EMF at 20 and 50 Hz can be neglected (*p* > 0.05).

These similar results in CT2A cell viability between the continuous exposure at short time and the intermittent exposure at long time stand out even more the frequency dependence. For this reason, we analyzed the effects of an EMF equal to the geomatic fields originated at the different frequencies of the Schuman resonance [23].

Hence, to study the frequency dependence, CT2A cells were exposed to a small EMF of 30 µT at the different frequencies of the Shuman resonance (Figure 1) [23]. According to our results, at the main frequency of 7.8 Hz and at the first harmonic 14 Hz, there is approximately a ±5% difference in cell viability with the control group which could be discarded (*p* > 0.05). However, it was found a significant decrease (*p* < 0.05) of around 30% in cell viability at 33 and at 26 Hz, which follows the reduction trend found in our studies at 100 µT EMF [5]. Additionally, our results show a cell viability decrease at 20, 39, 45, and 51 Hz (*p* < 0.05) with regard the control group (Helmholtz coils off), which seems to be related to the main cell viability decay at 26 and 33 Hz. In this way, according to our data (Figure 1), cell viability starts to decrease, reaching the most outstanding values at 26 and 33 Hz, after which the viability decrease starts to diminish. Nevertheless, this reduction pattern presents some setbacks at the frequencies of 20 and 51 Hz regarding our studies at short-term and long-term exposure to an EMF of 100 µT. However, it could be necessary to analyze in detail other frequencies in order to completely determine when the maximum decrease in cell viability is produced. Additionally, it is important to point out that tumoral cells can behave in a different way than nontumoral cell lines. In fact, we consider that difference one of the most remarkable results found in Reflex project [4] where it was found that, while ELF-EMF enhanced the proliferation of neuroblastoma cells (NB69), it didn’t affect the proliferation of lymphocytes (Human Lymphocytes from healthy donors). Therefore, the difference in cell proliferation found in our results should be in first instance studied in detail, using astrocytoma cell lines and based on our results, it is essential to study other parameters that could be related to the difference in cell viability after the exposure to ELF-EMF.

We decided to analyze the heat damage produced on CT2A exposed during 24 h to an EMF of 100 µT at 20, 30, and 50 Hz. The expression of heat effects was analyzed by the primary antibody anti-HSP90AB (Sigma Aldrich Ref: SAB4300541), which results in a cytoplasmic staining of the cell.

**Figure 7:**
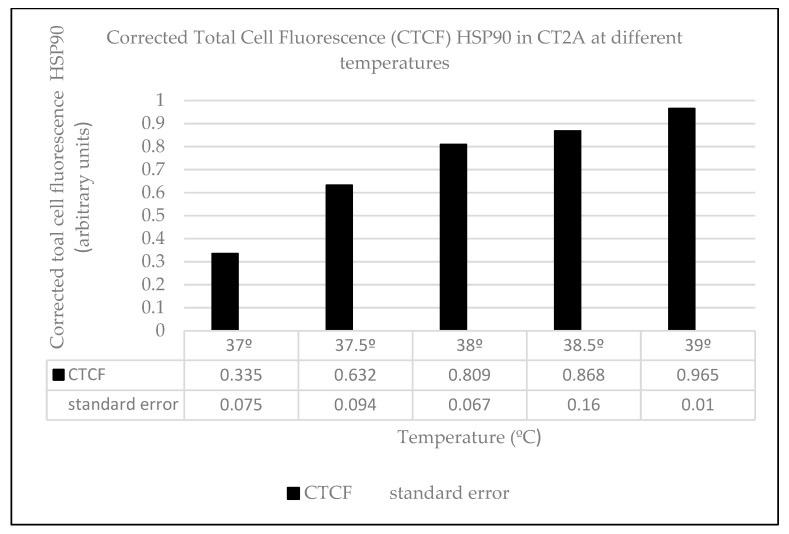
Corrected total cell fluorescence. Average corrected total cell fluorescence (CTCF) of HSP90 expression in CT2A maintained during 1 h and 30 min at 5% CO_2_ and different temperatures: 37, 37.5, 38, 38.5, and 39 °C.

However, according to our results, the exposure to an EMF of 100 µT at the different frequencies analyzed did not lead to heat damage on CT2A cells (Figure 4) since only basal expression of HSP90 was obtained (Figure 3B and Figure 4E,H,K). In fact, the quantification of the immunofluorescence signals of HSP90 in terms of corrected total cell fluorescence (CTCF) didn’t show difference (*p* > 0.05) between the frequencies analyzed (Figure 5) and the CT2A maintained during 1 h at 37 °C without exposure (Figure 7).

Additionally, to corroborate the results obtained in the immunofluorescence assay, we analyzed the HSP90 by immunoblot. Based on our Western blot (Figure 6) the difference in the total amount of HSP90 protein in the frequencies analyzed (20, 30, and 50 Hz) with respect the total amount of HSP90 protein in the control sample (Helmholtz Coils off, 0 Hz) was not significative (*p* > 0.05), nor was what we obtained before, in the immunofluorescence assay. Nevertheless, with our Western blot, we could observe a change in the pattern bands between 50 Hz and the rest of the frequencies (Figure 6). The upper band which appears in our results could be produced due to different modification as protein phosphorylation [27], protein ubiquitination [28], or protein sumoylation [29]. However, by analyzing together our results of the Western Blot and the immunofluorescence we don’t believe that the double band could be produced by protein phosphorylation, because it has been related in previous studies with nuclear HSP90 expression [27], and, in our case, it was always cytoplasmatic (Figure 4). On the other hand, we also don’t believe that the double band could correspond to protein ubiquitination, because that would have implied a pattern of multiple bands [28] instead of two (Figure 6).

Therefore, our results reveal that other crucial mechanisms should be related to the difference in cell viability produced at 30 Hz because the exposure to a 100 µT EMF at that frequency is under thermal effects.

In this sense, as it was previously investigated by other authors, one of those possible mechanism involved is the activation of the voltage-gated calcium channels (VGCCs) after the exposure to an EMF which are directly related to changes in calcium signaling [30]. On the other hand, the movement of Ca^2+^ ions through the transmembrane channels were also studied, taking into account the cyclotron resonance frequency (fc) of the ions, which is defined as $c=\frac{q}{2\pi m}$, with *q* being the charge of the ion and *m*, the mass [31]. Regarding that, different studies have been carried out, mainly with K^+^ and Ca^2+^ ions [32,33,34], even suggesting that the ion cyclotron resonance could be used as a tool in regenerative medicine [35].

## 4. Materials and Methods

### 4.1. Cell Culture

The mouse glioma cell line CT2A was maintained at physiological condition 37 °C and 5% CO_2_, in a Thermo Scientific incubator model 3111 series II. The CT2A cell line was grown in RPMI medium (Solmeglas w/l glutamine, w/25 mM Hepes) supplemented with 10% fetal bovine serum, 1% l-glutamine, and 1% streptomycin. The cells were seeded 24 h before the exposure in 35 mm cell culture dishes in a final concentration of 175,000 cells/mL for experiments lasting 24 h and at a final concentration of 37,500 cells/mL for experiment lasting 7 days.

### 4.2. Exposure System

**Figure 8:**
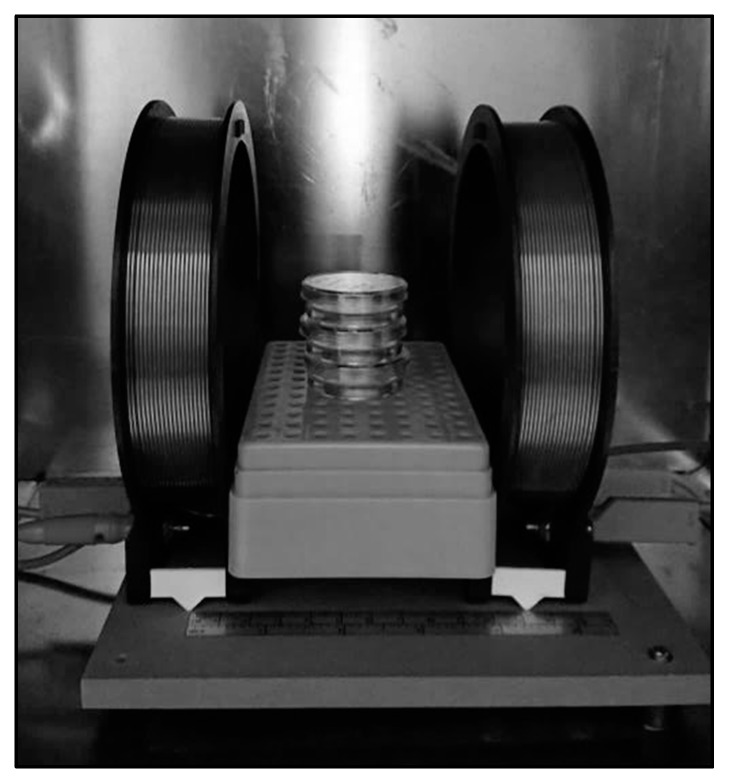
Exposure system constituted by a pair of Helmholtz Coils. The coils were placed inside a mu-metal box in a CO_2_ incubator.

The EMF was produced by a pair of Helmholtz coils of 19 turns and 12 cm of external radius, separated from each other by a distance equal to their radius. The pair of coils was placed inside the incubator in a mu-metal box, to avoid external fields. The culture dishes were placed in the middle of their axis, at 6 cm from each coil (Figure 8).

Each experiment was performed four times, using, in each of them, three culture dishes. Therefore, all the experiments were done with a total of twelve samples per frequency.

### 4.3. Methodology

To study the effect of natural EMF on cell viability, CT2A cells were exposed for 24 h to 30 µT EMF equal to the earth’s EMF [22] at the fundamental Schuman frequency of 7.8 Hz and the first harmonics: 14, 20, 26, 33, 39, 45, and 51 Hz [23].

On the other hand, to study the effect on cell viability of long-term and intermittent exposure to ELF-EMF, CT2A cells were exposed during 7 days to an intermittent EMF (12 h on/12 off pair of Helmholtz coils) of 100 µT at 20, 30, and 50 Hz.

Finally, to study the cellular damage and heat effect of ELF-EMF, CT2A cells were exposed during 24 h at 30 Hz to 100 µT equal the EMF stablished by the Council Recommendation 1999/519/EC for a frequency of 50 Hz [3].

The EMF generated in each experiment was first calculated theoretically according to Biot Savart Law and then set up by a power supply FAC 662B. The input signal was modulated with a function generator Frederiksen (10 Vpp, square signal). A LakeShore-Cryotronics 450 gaussemeter (LakeShore, Westerville, OH, USA) was used to measure the homogeneity of the EMF in the culture dishes obtaining mean values of 29.96 µT ± 0.94 standard error and 99.99 µT ± 6.81 standard error.

Control groups of non-exposed cells (Helmholtz coils off) were maintained in the same experimental condition for all the experiments done.

### 4.4. Cell Viability

The cell viability was measured by 2,3-Bis-(2-Methoxy-4-Nitro-5-Sulfophenyl)-2H-Tetrazolium-5-Carboxanilide (XTT) assay according to the instructions of the manufacturer (Cell Proliferation Kit XTT PanReac AppliChem) and the adaptations done before [6] (11). Briefly, after the exposure, the cells were incubated for 1 h at 37 °C and 5% CO_2_ with 114 µL of XTT reagent and 2 µL of activation solution per each 234 µL of medium. The absorbance was measured using a BioTek Instruments ELx800 absorbance microplate reader at a wavelength of 450 nm.

### 4.5. Immunofluorescence Assay

The expression of HSP90 as a marker of heat effect respectively was analyzed by indirect immunofluorescence. First, CT2A cells after the exposure were fixed with 4% paraformaldehyde (PFA) for 10 min at room temperature and incubated for 45 min in a blocking solution. After the fixation, CT2A cells were incubated for 1 h at room temperature with the primary antibody anti-HSP90AB1 (1:100 Sigma-Aldrich, St. Louis, MO, USA). Finally, the cells were incubated 1 h at room temperature with the secondary antibody anti-rabbit 546 (1:250, Hospital Puerta del Hierro). The nuclei of the cells were labelled with the fluorescence stay 4′,6-diamidino-2-phenylindole (DAPI 1:5000).

In order to differentiate properly the normal basal expression of the antibody anti-HSP90AB1 from the positive expression, CT2A cells were maintained during 1h and 30 min without EMF at different temperatures (37, 37.5, 38, 38.5, and 39 °C) and then immunolabeled with anti-HSP90. The range of temperatures was chosen from the fact that 37 °C is the normal temperature required for mammalian in vitro cultures and it has been shown in previous studies how an increase in 1 °C produced by RF exposure can have harmful effects [36].

The corrected total cell fluorescence (CTCF) of HSP90 in CT2A cell exposed to the different ELF was calculated as follows:

CTCF = Integrated Intensity of the cell − (Area of the cell × Mean of the background)(1)

The integrated intensity of the cell, the area of the cells and the mean of the background were obtained by using the program Image J.

### 4.6. Western Blot

The CT2A cells exposed during 24 h to the different ELFs-EMF (20, 30, and 50 Hz) and the control group of non-exposed CT2A cells (Helmholtz coils off) were lysed with RIPA buffer (50 mM of Tris, 1 mM of EDTA, 100 mM of NaCl, and 1% Triton) and mechanical scrapping. The cell suspensions were collected and centrifugated for 10 min, at 14,000 rpm.

After the centrifugation, in a 96 multi-well culture plate, we added 200 µL of a mixture of BCA solution buffer and CuSO4 to each 25 µL of supernatant, in order to quantify the protein HSP90 contained in the samples. The multi-well culture plate was kept in the dark for 30 min, at 37 °C. The total amount of HSP90 in each sample was obtained by measuring the absorbance at 562 nm in a Biobase reader.

Once it was quantified the HSP90 of each sample, an equal amount of 0.5 µg HSP90 protein was loaded together with a 4X BoltTM LDS buffer (Novex by Life Technologies, Carlsbad, CA, USA) to a acrylamide:bis-acrylamide gel in a mini Gel Tank (Invitrogen by ThermoFisher Scientific,). The gel was run for 1 h at room temperature at 100 V.

After the gel electrophoresis, the protein HSP90 was transferred from the gel to the membrane by using an IBlot 2 Gel Transfer Device (Invitrogen by ThermoFisher Scientific) and the membranes were immunoblotted. For that, to avoid nonspecific union, the membranes were first blocked with 5% infant milk powder during 1 h at room temperature and then incubated with the primary antibody anti-HSP90AB1 (1:500 Sigma-Aldrich) during 3 h at 4 °C. Finally, the membranes were incubated with the secondary antibody anti-rabbit 546 (1:20,000, Hospital Puerta del Hierro) during 45 min at room temperature and revealed with an ImageQuant LAS 500 chemiluminescence CCD camera (GE Healthcare).

### 4.7. Statistical Analysis

The statistical analysis in all the experiment was performed with Student’s *t*-test, at a confidence level of 95% (i.e., *p* < 0.05), to compare the following: (i) CT2A control group (Helmholtz coils off) vs. CT2A cells exposed during 24 h to a 30 µT of EMF at 7.8, 14, 20, 26, 33, 39, 45, and 51 Hz; (ii) CT2A control group (Helmholtz coils off) vs. CT2A cells exposed during 7 days to an intermittent 100 µT of EMF at 20, 30, and 50 Hz; (iii) CTCF of HSP90 expression in CT2A maintained at 37 °C without exposure vs. CT2A cells control group (Helmholtz coils off) and CT2A cells exposed during 24 h to a 100 µT of EMF at 20, 30, and 50 Hz; (iv) total amount of HSP90 protein in the immunoblotting of CT2A control group vs total amount of HSP90 protein in CT2A cells exposed during 24 h to a 100 µT EMF at 20, 30, and 50 Hz

The standard error was computed as follows:

(2) $$standard\;error=\frac{standard\;deviation}{\sqrt{N}}$$

where *N* is the number of samples (*N* = 12).

## 5. Conclusions

In conclusion, through our study of long-term exposure to a 100 µT EMF to different ELF and our study of the effect produced by a 30 µT EMF equal to the earth field [22] at the different frequencies of the Schuman resonance [23], we corroborated that frequency is determinant in cell viability. Moreover, we found that, contrary to what it has been proposed for a long time, the difference produced on cell viability is independent of the thermal effect (Figure 4).

However, several paradigms remain to be clarified about why EMFs at the frequency of 30 Hz produce a remarkable decrease in cell viability in CT2A cells. We selected CT2A cells for our research for two main reason: First, we aimed to study the effect of EMF on a tumoral cell line, and second, we used them in previous studies [5], where we found viability dependence on frequency which we want to corroborate. Therefore, based on the results obtained until the date, it could be thought that frequency seems to modulate the response of the cells, including difference in cell viability and in the state of HSP90. Thus, it could be related to the ion cyclotron frequency. Nevertheless, to test that hypothesis, it is necessary to extend the studies to nontumoral cell lines, as well as it is important to analyze other cell viability markers, the nuclear damage produced, and the measurement of the amount of intracellular Ca^2+^ in CT2A cells after the exposure among others crucial parameters.

## Abbreviations

MDPI	Multidisciplinary Digital Publishing Institute
DOAJ	Directory of open access journals
TLA	Three letter acronym
LD	Linear dichroism

## References

[1] Korada U. (1989). Introduction to Engineering Electromagnetic Fields. Advanced Series in Electrical and Computer Engineering.

[2] WHO What Are Electromagnetic Fields?. https://www.who.int/peh-emf/about/WhatisEMF/en/.

[3] Council Recommendation 1999/519/EC Council Recommendation of 12 July 1999 on the Limitation of Exposure of the General Public to Electromagnetic Fields (0 Hz to 300 GHz). https://eur-lex.europa.eu/LexUriServ/LexUriServ.do?uri=OJ:L:1999:199:0059:0070:EN:PDF.

[4] Adlkofer F., Tauber R.W., Rüdiger H.G., Wobus A.M., Trillo A., Leszczynski D., Kolb H.A., Bersani F., Lagroye I., Kuster N. (2004). Risk Evaluation of Potential Environmental Hazards from Low Energy Electromagnetic Field Exposure Using Sensitive in vitro Methods. Final Report of a Project Funded by the European Union under the Programme Quality of Life and Management of Living Resources.

[5] García-Minguillán López O., Jiménez Valbuena A., Maestú Unturbe C. (2019). Significant Cellular Viability Dependence on Time Exposition at ELF-EMF and RF-EMF In Vitro Studies. Int. J. Environ. Res. Public Health.

[6] Walleczek J. (1992). Electromagnetic field effects on cells of the immune system: The role of calcium signaling. FASEB J..

[7] Grassi C., D’Ascenzo M., Torsello A., Martinotti G., Wolf F., Cittadini A., Azzena G.B. (2004). Effects of 50 Hz electromagnetic fields on voltage-gated Ca^2+^ channels and their role in modulation of neuroendocrine cell proliferation and death. Cell Calc..

[8] Jeong J.H., Kum C., Choi H.J., Park E.S., Sohn U.D. (2006). Extremely low frequency magnetic field induces hyperalgesia in mice modulated by nitric oxide synthesis. Life Sci..

[9] Lorich D.G., Brighton C.T., Gupta R., Corsetti J.R., Levine S.E., Gelb I.D., Seldes R., Pollack S.R. (1998). Biochemical pathway mediating the response of bone cells to capacitive coupling. Clin. Orthop. Relat. Res..

[10] Piacentini R., Ripoli C., Mezzogori D., Azzena G.B., Grassi C. (2008). Extremely low-frequency electromagnetic fields promote in vitro neurogenesis via upregulation of Ca(v)1-channel activity. J. Cell. Physiol..

[11] Brini M., Calì T., Ottolini D., Carafoli E. (2013). Intracellular calcium homeostasis and signaling. Met. Ions Life Sci..

[12] Brini M., Ottolini D., Calì T., Carafoli E. (2013). Calcium in health and disease. Met. Ions Life Sci..

[13] Martin L.P. (2013). Electromagnetic fields act via activation of voltage-gated calcium channels to produce beneficial or adverse effects. J. Cell. Mol. Med..

[14] Luukkonen J., Höytö A., Sokka M., Liimatainen A., Syväoja J., Juutilainen J., Naarala J. (2017). Modification of p21 level and cell cycle distribution by 50 Hz magnetic fields in human SH-SY5Y neuroblastoma cells. Int. J. Radiat. Biol..

[15] Qinlong M., Ping D., Gang Z., Chuan L., Lei Z., Zhou Z., Xue L., Min L., Min Z., Zhengping Y. (2014). Extremely Low-Frequency Electromagnetic Fields Affect Transcript Levels of Neuronal Differentiation-Related Genes in Embryonic Neural Stem Cells. PLoS ONE.

[16] Solek P., Majchrowicz L., Bloniarz D., Krotoszynska E., Koziorowski M. (2017). Pulsed or continuous electromagnetic field induce p53/p21-mediated apoptotic signaling pathway in mouse spermatogenic cells in vitro and thus may affect male fertility. Toxicology.

[17] Akdag M.Z., Dasdag S., Uzunlar A.K., Ulukaya E., Oral A.Y., Çelik N., Akşen F. (2013). Can safe and long-term exposure to extremely low frequency (50 Hz) magnetic fields affect apoptosis, reproduction, and oxidative stress?. Int. J. Radiat. Biol..

[18] Liu Y.X., Tai J.L., Li G.Q., Zhang Z.W., Xue J.H., Liu H.S., Zhu H., Cheng J.D., Liu Y.L., Li A.M. (2012). Exposure to 1950-MHz TD-SCDMA electromagnetic fields affects the apoptosis of astrocytes via caspase-3-dependent pathway. PLoS ONE.

[19] Lantow M., Lupke M., Frahm J., Mattsson M.O., Kuster N., Simko M. (2006). ROS release and Hsp70 expression after exposure to 1800 MHz radiofrequency electromagnetic fields in primary human monocytes and lymphocytes. Radiat. Environ. Biophys..

[20] Falone S., Santini S., Cordone V., Cesare P., Bonfigli A., Grannonico M., Di Emidio G., Tatone C., Cacchio M., Amicarelli F. (2017). Power frequency magnetic field promotes a more malignant phenotype in neuroblastoma cells via redox-related mechanisms. Sci. Rep..

[21] Lee H.C., Hong M.N., Jung S.H., Kim B.C., Suh Y.J., Ko Y.G., Lee Y.S., Lee B.Y., Cho Y.G., Myung S.H. (2015). Effect of extremely low frequency magnetic fields on cell proliferation and gene expression. Bioelectromagnetics.

[22] Finlay C.C., Maus S., Beggan C.D., Bondar T.N., Chambodut A., Chernova T.A., Chulliat A., Golovkov V.P., Hamilton B., Hamoudi M. (2010). International Geomagnetic Reference Field: The eleventh generation. Geophys. J. Int..

[23] Votis C.I., Tatsis G., Christofilakis V., Chronopoulos S.K., Kostarakis P., Tritakis V., Repapis C. (2018). A new portable ELF Schumann resonance receiver: Design and detailed analysis of the antenna and the analog front-end. EURASIP J. Wirel. Commun. Netw..

[24] Akbarnejad Z., Eskandary H., Vergallo C., Nematollahi-Mahani S.N., Dini L., Darvishzadeh-Mahani F., Ahmadi M. (2017). Effects of extremely low-frequency pulsed electromagnetic fields (ELF-PEMFs) on glioblastoma cells (U87). Electromagn. Biol. Med..

[25] Kim S., Im W.S., Kang L., Lee S.T., Chu K., Kim B.I. (2008). The application of magnets directs the orientation of neurite outgrowth in cultured human neuronal cells. J. Neurosci. Methods.

[26] Koziorowska A., Romerowicz-Misielak M., Sołek P., Koziorowski M. (2018). Extremely low frequency variable electromagnetic fields affect cancer and noncancerous cells in vitro differently: Preliminary study. Electromagn. Biol. Med..

[27] Dagar M., Singh J.P., Dagar G., Tyagi R.K., Bagch G. (2019). Phosphorylation of HSP90 by protein kinase A is essential for the nuclear translocation of androgen receptor. J. Biol. Chem..

[28] Morales J.L., Perdew G.H. (2007). Carboxyl Terminus of hsc70-Interacting Protein (CHIP) Can Remodel Mature Aryl Hydrocarbon Receptor (AhR) Complexes and Mediate Ubiquitination of Both the AhR and the 90 kDa Heat-Shock Protein (hsp90) in Vitro. Biochemistry.

[29] Mayer M.P., Le Breton L. (2015). Hsp90: Breaking the Symetry. Mol. Cell.

[30] Martin L.P. (2015). Scientific evidence contradicts findings and assumptions of Canadian Safety Panel 6: Microwaves act through voltage-gated calcium channel activation to induce biological impacts at non-thermal levels, supporting a paradigm shift for microwave/lower frequency electromagnetic field action. Rev. Environ. Health.

[31] Liboff R.A. (1985). Geomagnetic cyclotron resonance in living cells. J. Biol. Phys..

[32] Blackman C.F., Blanchard J.P., Benane S.G., House D.E. (1994). Empirical test of an ion parametric resonance model for magnetic field interactions with PC-12 cells. Bioelectromagnetics.

[33] Lerchl A., Reiter R.J., Howes K.A., Nonaka O.N., Stokkan K.A. (1991). Evidence that extremely low frequency Ca-cyclotron resonance depresses pineal melatonin synthesis in vitro. Neurosci. Lett..

[34] Liboff R.A. (2019). Ion cyclotron resonance: Geomagnetic strategy for living systems?. Electromagn. Biol. Med..

[35] Lisi A., Ledda M., de Carlo F., Pozzi D., Messina E., Gaetani R., Chimenti I., Barile L., Giacomello A., D’Emilia E. (2008). Ion cyclotron resonance as a tool in regenerative medicine. Electromagn. Biol. Med..

[36] American Conference of Government Industrial Hygienists (1996). Threshold Limit Values for Chemical Substances and Physical Agents and Biological Exposure Indices.

